# Gut microbiota-derived metabolites in inflammatory diseases based on targeted metabolomics

**DOI:** 10.3389/fphar.2022.919181

**Published:** 2022-09-27

**Authors:** Hui Xu, Li-Bin Pan, Hang Yu, Pei Han, Jie Fu, Zheng-Wei Zhang, Jia-Chun Hu, Xin-Yu Yang, Adili Keranmu, Hao-Jian Zhang, Meng-Meng Bu, Jian-Dong Jiang, Yan Wang

**Affiliations:** State Key Laboratory of Bioactive Substance and Function of Natural Medicines, Institute of Materia Medica, Chinese Academy of Medical Sciences/Peking Union Medical College, Beijing, China

**Keywords:** inflammatory diseases, amino acid metabolites, tryptophan, phenylalanine, histidine, gut microbiota, LC-MS/MS

## Abstract

The gut microbiota plays an important role in inflammatory diseases. Metabolites in the three metabolic pathways of tryptophan (Trp), histidine (His), and phenylalanine (Phe) can affect various inflammatory conditions, such as obesity, diabetes, arthritis, colitis, atherosclerosis, and neuroinflammation. We established an LC–MS/MS method to measure 17 metabolites—Trp, 3-indole-acetic acid (Iaa), 3-indole-lactate (Ila), 3-indole-propionic acid (Ipa), 3-indole formaldehyde (Iald), kynurenine (Kn), kynurenic acid (Kyna), 3-Hydroxyanthranilic acid (3-Haa), His, 3-methylhistidine (3-Mhis), histamine (Hist), imidazole propionic acid (Imp), 4-imidazoacetic acid (Imaa), urocanic acid (Ua), Phe, phenylethylamine (Pea), and hippuric acid (Ha)—in the three metabolic pathways. The method exhibited high sensitivity and good selectivity, linearity, accuracy, precision, stability; and recovery rate; all met the requirements of biological sample analysis. By establishing a rheumatoid arthritis (RA) model of Sprague–Dawley rats and performing 16S rRNA sequencing on their feces, it was found that there was dysbiosis, including changes in phylum level, genus level, and *α* biodiversity of gut bacteria. The contents of the microbiota metabolites Iaa and Ipa in the model group were significantly decreased, and those of Iald, Kn, Kyna, Ha, and Imp were significantly increased. The common therapeutic drugs *Tripterygium* glycosides, total glucosides of peony, and their main active ingredients were screened by *in vitro* incubation with gut bacteria: it was found that *Tripterygium* glycosides and their active ingredients could lead to a variation in metabolites in the Trp and Phe pathways. Total glucosides and active components of peony could lead to a variation in metabolites in the Phe pathway of the gut microbiota.

## Graphical Abstract

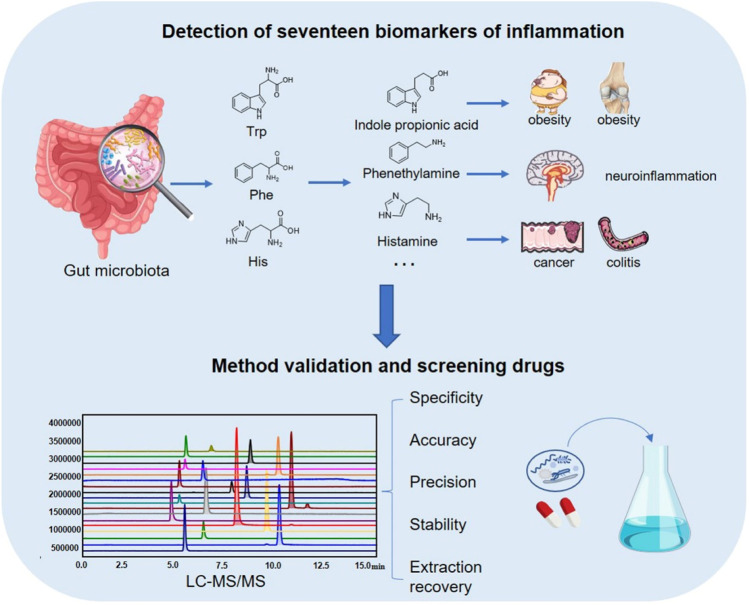

## Introduction

Inflammation is the body’s defense response to stimuli such as infection and tissue damage, including acute and chronic inflammation. Acute inflammation is of short duration, while chronic inflammation is associated with the immune system and underlies the progression of diseases such as obesity (Truax et al., 2018), diabetes (Kanazawa et al., 2021), arthritis (Vadell et al., 2020), inflammatory bowel disease (Silverberg et al., 2005), cardiovascular diseases (Ridker et al., 2019), neurological disease (Jensen et al., 2019), cancer (Singh N. et al., 2019), and autoimmune disease (Cai et al., 2019). These inflammatory diseases seriously affect human health and quality of life. Although they are also influenced by important factors other than inflammation, inhibition of inflammation often improves the clinical symptoms of these diseases (Ridker et al., 2017; Jensen et al., 2019; Vadell et al., 2020; Chicco et al., 2021). However, the mode of action of inflammation is very complex and has not yet been fully elucidated.

There are more than 100 trillion microorganisms in the human intestine, collectively referred to as the “gut microbiota”, which play a very important role in maintaining human health and are considered the “invisible organ” of the human body (Collins and Patterson, 2020; Pan et al., 2020; Wang et al., 2021). In recent years, the pathogenesis of inflammation-related diseases has been shown to be closely related to the gut microbiota, including intestinal inflammation, inflammation of organs other than the gut, and systemic inflammation (Yang et al., 2020b; Zhang X. et al., 2021; He et al., 2021; Shang et al., 2021). Targeting the gut microbiota for the treatment of inflammatory diseases has great potential. The mechanism of gut microbiota involvement in the occurrence and development of inflammatory diseases is very complex, and research on how intestinal metabolites and the host interact to affect diseases is a hot topic. The influence of gut metabolites on the inflammation of organs other than the gut and on systemic inflammation is mainly because the metabolites can be absorbed into the blood by intestinal epithelial cells to enter systemic circulation.

We selected the metabolic pathways of three essential amino acids—tryptophan (Trp), phenylalanine (Phe), and histidine (His)—which are closely related to inflammation. The metabolism of Trp by bacteria is mainly divided into two pathways: indole and kynurenine. A portion of Trp is metabolized into Iaa, Ipa, Ila, and Iald, which are released into the systemic circulation. Trp is metabolized by *Lactobacilli* in the gut through aromatic amino acid aminotransferase (ArAT) and indole lactate dehydrogenase (ILDH) to the intermediate product indolepyruvate, which further generates Iald. *Peptostreptococcus* bacteria such as *P. anaerobius* and *P. stomatis* that contain the phenyllactate dehydratase gene cluster (fldAIBC) in the gut can metabolize Trp into Iaa, Ipa, and Ila (Wlodarska et al., 2017; Roager and Licht, 2018). The other portion of Trp is metabolized into Kn by indoleamine 2,3-dioxygenase 1 (IDO1) and Kyna; Kn is further converted into 3-Haa through hydroxylation (Agus et al., 2018). Phe is produced in the gut microbiota by *Morganella morganii* decarboxylase to phenylethylamine. It is first metabolized into trans-cinnamic acid, which is further metabolized to hippuric acid. His is produced by histidine decarboxylase (HDC) to generate histamine, which is further oxidized to imidazole-4-acetic acid. Histamine is a well-known proinflammatory factor that induces different immune cells to produce inflammatory mediators and cytokines (Branco et al., 2018). His is degraded by histidine ammonia lyase (HAL) or histidase to urocanic acid, which is reduced to imidazole propionic acid by urocanic acid reductase (Acuña et al., 2021). These amino acid metabolites are closely related to inflammatory diseases. The Trp metabolite Iald can activate aromatic hydrocarbon receptor (AHR) and induce the expression of interleukin-22 (IL-22) to improve the intestinal barrier and alleviate colitis in mice (Teng et al., 2018). The His metabolite histamine can modulate NLRP6 inflammasome signaling and downstream antimicrobial peptide secretion, promote interleukin-18 (IL-18) secretion from intestinal epithelial cells, and shape the gut microenvironment through the metabolite-inflammasome-antimicrobial peptide axis (Levy et al., 2015). Phe metabolites are related to neuritis. Therefore, the search for inflammation-related amino acid microbiota metabolites is of great significance for elucidating the mechanisms of various inflammatory diseases and screening drugs.

Although many LC–MS/MS analytical methods for determining Trp metabolites have been reported in the literature, there are few analytical methods for His and Phe metabolites, and there is still no simultaneous analytical method for the determination of the 17 metabolites on three metabolic pathways of Trp, His, and Phe (Fuertig et al., 2016; Wang et al., 2019). We constructed a simple and rapid LC–MS/MS analytical method with sufficient sensitivity to detect intestinal content; this method can be applied to various inflammatory diseases as targeted metabolomics.

## Materials and methods

### Reagents and materials

Trp, indole acetic acid (Iaa), indole propionic acid (Ipa), indole lactic acid (Ila), kynurenine (Kn), kynurenic acid (Kyna), His, histamine (Hist), urocanic acid (Ua), Phe, hippuric acid (Ha), acetaminophen (IS), triptolide (TL), celastrol (CSL), wilforine (WR), wilforlide A, and triptonide (TN), paeoniflorin, albiflorin std, and benzoylpaeoniflorin were purchased from Solarbio Scientific Ltd. (Beijing, China). 3-hydroxyanthranilic acid (3-Haa) and indole formaldehyde (Ilad) were purchased from Yuanye Biotechnology Co., Ltd. (Shanghai, Beijing). Imidazole propionic acid (Imp), imidazole-4-acetic acid (Imaa), 3-methyl-l-histidine (3-Mhis), and phenethylamine (Pea) were purchased from Aladdin Biochemical Technology Co., Ltd. (Shanghai, China). The purities of all the reference standards were greater than 98%. *Tripterygium wilfordii* polyglycoside tablets (henceforth referred to as “*Tripterygium* glycosides”) were purchased from Zhejiang Deen Pharmaceutical Co., Ltd. (Hangzhou, China). Total glucosides of white paeony capsules (henceforth referred to as “total glucosides of peony”) were purchased from Ningbo Lihua Pharmaceutical Co., Ltd. Formic acid (100%), complete Freund’s adjuvant, and isoflurane were purchased from Merck (Darmstadt, Germany). Acetonitrile and methanol were purchased from Fisher Scientific (HPLC grade, Fairlawn, United States). Deionized distilled water was purchased from Hangzhou Wahaha Group Co., Ltd. (Hangzhou, China). TNF-α, IL-1β, and IL-6 kits were purchased from Nanjing Jiancheng Bioengineering Institute (Nanjing, China). Anaerobic culture medium was purchased from Qingdao Hope Bio-Technology Co., Ltd. (Qingdao, China).

### Instruments

A high-performance liquid chromatography–mass spectrometry (LC–MS/MS 8060, Shimadzu, Japan) instrument was utilized. A vortex mixer (VortexGenie2, United States), a small benchtop high-speed centrifuge (Eppendorf 5418, Germany), a 1–14 small benchtop high-speed centrifuge (D-37520, Sigma, Germany), an analytical balance (XS1050U, Mettler - Toledo, Switzerland), an incubator shaker (LYZ-100, Shanghai Longyue Co., Ltd., China), and a nitrogen vaporizer (MD 200-2, Hangzhou Diansheng Instrument Co., Ltd., China) were utilized.

### Animals

Sprague–Dawley rats (180–220 g, 6–8 weeks, male) were provided by Beijing Huafukang Biotechnology Co., Ltd. (Beijing, China). All animals had free access to food and water. The temperature was maintained at 22–24°C with a light/dark cycle of 12 h and a relative humidity of 40–60%. Fresh stool samples were collected in sterile nitrogen-filled zip-lock bags and kept at −70°C.

### LC–MS/MS conditions

Analysis was performed using a liquid chromatography-tandem mass spectrometer LC–MS/MS 8060 (Shimadzu, Japan) equipped with an ESI source. Separation was performed using a C18 column (250 mm × 4.6 mm × 5 μm, SVEA, Sweden). The flow rate was 0.4 ml/min, and the column temperature was maintained at 25°C. The mobile phases were formic acid: water (0.1:100, v/v) as mobile phase A, and methanol:acetonitrile (1:1) and 0.1% formic acid as mobile phase B. The binary gradient elution conditions were: (A:B): 0.01 min–5 min, 70:30→5:95; 5–8 min, 5:95; 8.01 min–16 min, 70:30. Detection was performed using multiple reaction monitoring (MRM) in positive mode, and the optimized MRM parameters for each compound are shown in Table 1. The mass condition parameters were set as: nebulizer gas, 3 L/min; drying gas, 10.0 L/min; heating gas, 10.0 L/min; interface temperature, 300°C; collision-induced dissociation (CID) gas, 230 kPa; DL temperature, 250°C; thermal block temperature, 400°C; interface voltage, −4.5 kV. The autosampler was kept at 4°C.

**TABLE 1:** Optimized multiple reaction monitoring (MRM, positive) and mass spectrometry (MS) conditions.

Analyte	Formula	MW	Precursor Ion (m/z)	Quantification (m/z)	Quantifier (m/z)	Q1 CE (volt)	Q2 CE (volt)	Q3 CE (volt)
Hippuric acid (Ha)	C_9_H_9_NO_3_	179.17	179.75	105.05	77.00	−21.0	−13.0	−10.0
Imidazole-4-acetic acid (Imaa)	C_5_H_6_N_2_O_2_	126.11	127.00	54.00	81.10	−13.0	−30.0	−19.0
Phenylethylamine (Pea)	C_8_H_11_N	121.18	122.05	77.05	105.05	−12.0	−28.0	−13.0
3-Methylhistidine (3-Mhis)	C_7_H_11_N_3_O_2_	169.18	170.00	94.95	109.10	−10.0	−30.0	−29.0
Urocanic acid (Ua)	C_6_H_6_N_2_O_2_	138.12	139.00	93.00	121.10	−15.0	−22.0	−15.0
Imidazole propionic acid (Imp)	C_6_H_8_N_2_O_2_	140.14	140.95	81.10	123.05	−10.0	−22.0	−30.0
Tryptophan (Trp)	C_11_H_12_N_2_O_2_	204.23	205.00	189.20	146.10	−16.0	−10.0	−17.0
Phenylalanine (Phe)	C_9_H_11_NO_2_	165.19	166.00	120.10	103.00	−12.0	−13.0	−11.0
Histidine (His)	C_6_H_9_N_3_O_2_	155.15	156.00	110.05	93.00	−10.0	−15.0	−10.0
Histamine (Hist)	C_5_H_9_N_3_	111.15	112.05	95.10	83.04	−10.0	−15.0	−18.0
Indole formaldehyde Iald)	C_9_H_7_NO	145.16	145.95	91.05	118.00	−16.0	−26.0	−13.0
Kynurenic acid (Kyna)	C_10_H_7_NO_3_	189.17	189.90	144.00	171.96	−13.0	−19.0	−13.0
Indole lactic acid (Ila)	C_11_H_11_NO_3_	205.21	205.90	130.00	117.89	−15.0	−33.0	−20.0
Indole acetic acid (Iaa)	C_10_H_9_NO_2_	175.18	176.00	130.00	77.03	−12.0	−14.0	−20.0
Indole propionic acid (Ipa)	C_11_H_11_NO_2_	189.20	189.95	130.15	55.01	−10.0	−17.0	−19.0
Kynurenine (Kn)	C_10_H_12_N_2_O_3_	208.22	209.10	192.00	146.00	−15.0	−11.0	−18.0
3-Hydroxyanthranilic acid (3-Haa)	C_7_H_7_NO_3_	153.14	153.90	136.00	108.00	−18.0	−14.0	−22.0

### Standard solutions and sample preparation

Trp, Kn, Phe, His, Hist, 3-Mhis, Ua, and Pea were dissolved in 2% aqueous formic acid, and Iaa, Ila, Ipa, Iald, Kyna, 3-Haa, Imp, Ha, and Imaa were dissolved in methanol containing 2% formic acid, all prepared to 1 mg/ml, for use. The IS was dissolved in methanol containing 2% formic acid to prepare 1 mg/ml. The lower limits of quantitation and detection were determined by serial dilutions of the non-matrix stock solution in methanol containing 2% formic acid. After mixing the intestinal contents of 20 normal rats, anaerobic culture medium at a 1:20 (weight: volume) ratio as matrix was added. Calibration standards were prepared by spiking the mixed stocking solutions at a volume ratio of 1:9, then adding at a volume ratio of 1:3 of methanol containing IS (1ug/ml) and 2% formic acid (100, 200, 500, 1000, 2000, 8000, 10,000, and 12500 ng/ml for Trp, Phe, His, and Imp; 20, 40, 100, 200, 400, 1000, 2000, and 2500 ng/ml for other compounds); 5 µL of the supernatant was taken for injection after centrifugation at 12,000 rpm for 10 min at 4°C. Low concentration QC, medium concentration QC, and high concentration QC were prepared by spiking the mixed stocking solutions in matrix at a volume ratio of 1:9 (200, 2000, and 10,000 ng/ml for Trp, Phe, His, and Imp; 40, 400, and 2000 ng/ml for other compounds). The specific steps are shown in Supplementary Figure S1. For sample preparation, added methanol solution containing IS (1 μg/ml) and 2% formic acid at a volume ratio of 1:3.5 µL of the supernatant was taken for injection after centrifugation at 12,000 rpm for 10 min at 4°C.

### Method validation

#### Specificity and residue

Specificity was obtained by comparing the chromatogram of the standard added to the matrix to the chromatogram of the matrix. After five consecutive injections of high concentration quality control (HQC), the residue was judged by the response of the injection blank solvent.

#### Accuracy and precision

Accuracy was assessed on samples of known analytes, using three batches of quality control samples with high, medium, and low concentrations, five samples per concentration, and expressed as measured value/true value*100%.

Precision was repeated for three consecutive days using three batches of high, medium, and low concentrations of quality control samples, with five samples per concentration; the precision was expressed as the relative standard deviation.

#### Linear range and lower limit of quantitation

A non-zero calibration curve was established by plotting the peak area ratio of analyte to internal standard (Y) versus the nominal concentration of compound added to the matrix sample (X). The correlation coefficient (*R* ^2^) was used to assess linearity and was fitted with a weighting factor of 1/X. The linear range was accepted when the relative error of the calibrator was within ±15% of the theoretical concentration.

The lower limit of quantification was determined by continuous dilution of the standard solution. A signal-to-noise (S/N) ratio greater than 3 for each compound is the LLOD, and an S/N greater than 10 is the instrument’s LLOQ.

#### Stability

Stability was assessed using spiked samples (LQC, MQC, and HQC), five samples of each concentration, placed at 4°C for 12 h before or after sample treatment, or by repeated freeze–thaw cycles at −20°C before treatment three times. Stability was calculated by the ratio of the concentration of each compound before treatment to the concentration of the corresponding sample after treatment. Data in the 85–115% range are considered stable.

#### Extraction recovery

Extraction recovery is the ratio of sample concentration after extraction/before extraction; data in the 85–115% range are considered acceptable.

#### Matrix effect

Matrix effect = (concentration of standard-spiked samples – concentration of matrix)/standards free from matrix.

### Establishment of a rat model of rheumatoid arthritis

Ten Sprague-Dawley rats (male, 6–8 weeks) were randomly divided into two groups—a blank control group and a model group—with five animals in each group. After isoflurane anesthesia, 100 µL of normal saline was injected into the soles of the right feet of the blank group, and 100 µL of complete Freund’s adjuvant was injected into the soles of the right feet of the model group. After 21 days, the exact same procedure was repeated. After 25 days, the rats were weighed, fresh feces and blood were collected, and the rats were sacrificed by removing their cervical vertebrae. The contents of intestinal bacteria were collected, and the spleen was weighed.

### 16S rRNA sequencing of feces

#### The DNA extraction

DNA was extracted using PowerSoil DNA Isolation Kit (MoBio Laboratories, Carlsbad, CA) following the manual. Purity and quality of the genomic DNA were checked on 1% agarose gels and a NanoDrop spectrophotometer (Thermo Scientific).

### PCR amplification

The V3-4 hypervariable region of bacterial 16S rRNA gene were amplified with the primers 338F (ACT​CCT​ACG​GGA​GGC​AGC​AG) and 806R (GGACTACHVGGGTWTCTAAT). For each fecal sample, a ten-digit barcode sequence was added to the 5’ end of the forward and reverse primers (provided by Allwegene Company, Beijing). The PCR was carried out on a Mastercycler Gradient (Eppendorf, Germany) using 25 µL reaction volumes, containing 12.5 μL KAPA 2G Robust Hot Start Ready Mix, 1 µL Forward Primer (5 µM), 1 µL Reverse Primer (5 µM), 5 µL DNA (total template quantity is 30 ng), and 5.5 µL H_2_O. Cycling parameters were 95°C for 5 min, followed by 28 cycles of 95°C for 45 s, 55°C for 50 s, and 72°C for 45 s, with a final extension at 72°C for 10 min. The PCR products were purified using a Agencourt AMPure XP Kit.

#### High throughput sequencing

Deep sequencing was performed on the Miseq platform at Allwegene Company (Beijing). After the run, image analysis, base calling, and error estimation were performed using Illumina Analysis Pipeline Version 2.6.

#### Data analyses

The raw data were first screened: sequences were removed from consideration if they were shorter than 230 bp, had a low-quality score (≤20), contained ambiguous bases, or did not exactly match the primer sequences and barcode tags. Qualified reads were separated using the sample-specific barcode sequences and trimmed with Illumina Analysis Pipeline Version 2.6. The dataset was then analyzed using QIIME. The sequences were clustered into operational taxonomic units (OTUs) at a similarity level of 97%, to generate rarefaction curves and calculate the richness and diversity indices (including Chao 1, Observed_ species, PD_ whole_ trees, and Shannon indices). Histogram analysis of *α* biodiversity was performed using GraphPad Prism 8. The Ribosomal Database Project (RDP) Classifier tool was used to classify all sequences into different taxonomic groups. To examine the similarity between different samples, clustering analyses and PCA were used based on the OTU information from each sample using R. The evolution distances between microbial communities from each sample were calculated using the Bray Curtis algorithms and represented as an unweighted pair group method with an arithmetic mean (UPGMA) clustering tree describing the dissimilarity (1-similarity) between multiple samples. A Newick-formatted tree file was generated using this analysis. To compare the membership and structure of communities in different samples, histogram analysis of changes in phylum level relative abundance was performed using GraphPad Prism 8. Changes in relative abundance at genus level were shown as a heatmap, which was processed in R.

### Screening of drugs for RA *in vitro*

The intestinal contents of normal Sprague-Dawley rats were obtained, weighed, added to anaerobic culture medium at a 1:20 (w/v) ratio, preincubated for half an hour at 37°C and 200 rpm, and 10 µL of the target drugs or compounds solutions (1 mg/ml and 10 mg/ml) was added to 990 µL of anaerobic culture medium containing gut microbiome to achieve final concentrations of 10 μg/ml (low dose) and 100 μg/ml (high dose). The drug or compound solutions were added in advance, and anaerobic culture medium containing gut microbiome was added during nitrogen purging. Chosen drugs and compounds includes *Tripterygium* glycosides, triptolide, celastrol, wilforine, wilforlide A, triptonide, total glucosides of peony, paeoniflorin, albiflorin std, and benzoylpaeoniflorin. For total glucosides of peony, the contents of the capsule were weighed and dissolved with methanol. For *Tripterygium* glycosides, we first crushed the tablets with a mortar, then weighed them, added methanol, and dissolved them by ultrasound. All dissolved drug or compound solutions should be filtered by microporous membranes (0.22 um). We first added the solution filtered by the microporous membrane to the EP tube, then added intestinal bacterial incubation solution. For the control group, exactly the same procedure was repeated, except that the compound and drug solutions were replaced with methanol solution. After incubating for 12 h at 37°C and 200 rpm, the samples were immediately analyzed according to “Standard Solutions and Sample Preparation”.

Data sets were obtained from LC-MS. Modules for quantitative analysis were selected by SIMCA (MKS Umetrics, Sweden). Automatic construction simulation was then performed on adjusted parameters and number groups. Finally, data from all groups were scored and a principal component analysis (PCA) score graph was acquired.

Imported data sets were obtained from LC-MS into R software. We read the data set, defined the colors of the heatmap, modified the legend size and scope, and performed normalization of datasets which controlled ranges from −1 to 1. Finally, the heatmap was acquired.

### Statistical analysis

Data analysis was performed using GraphPad Prism 8. A two-sided *t* test was used; *p* < 0.05 was considered statistically significant. Heatmaps were processed in R, with blue representing lower levels and red representing higher levels. The PCA graph was processed using SIMCA (MKS Umetrics, Sweden), and the data were normalized before plotting.

## Results

### Method development

To establish targeted metabolomics methods, we optimized mass spectrometry, chromatographic conditions, and sample preparation in order to obtain optimal sensitivity, separation, and quantitative accuracy.

#### Mass spectrometric conditions

**FIGURE 1:**
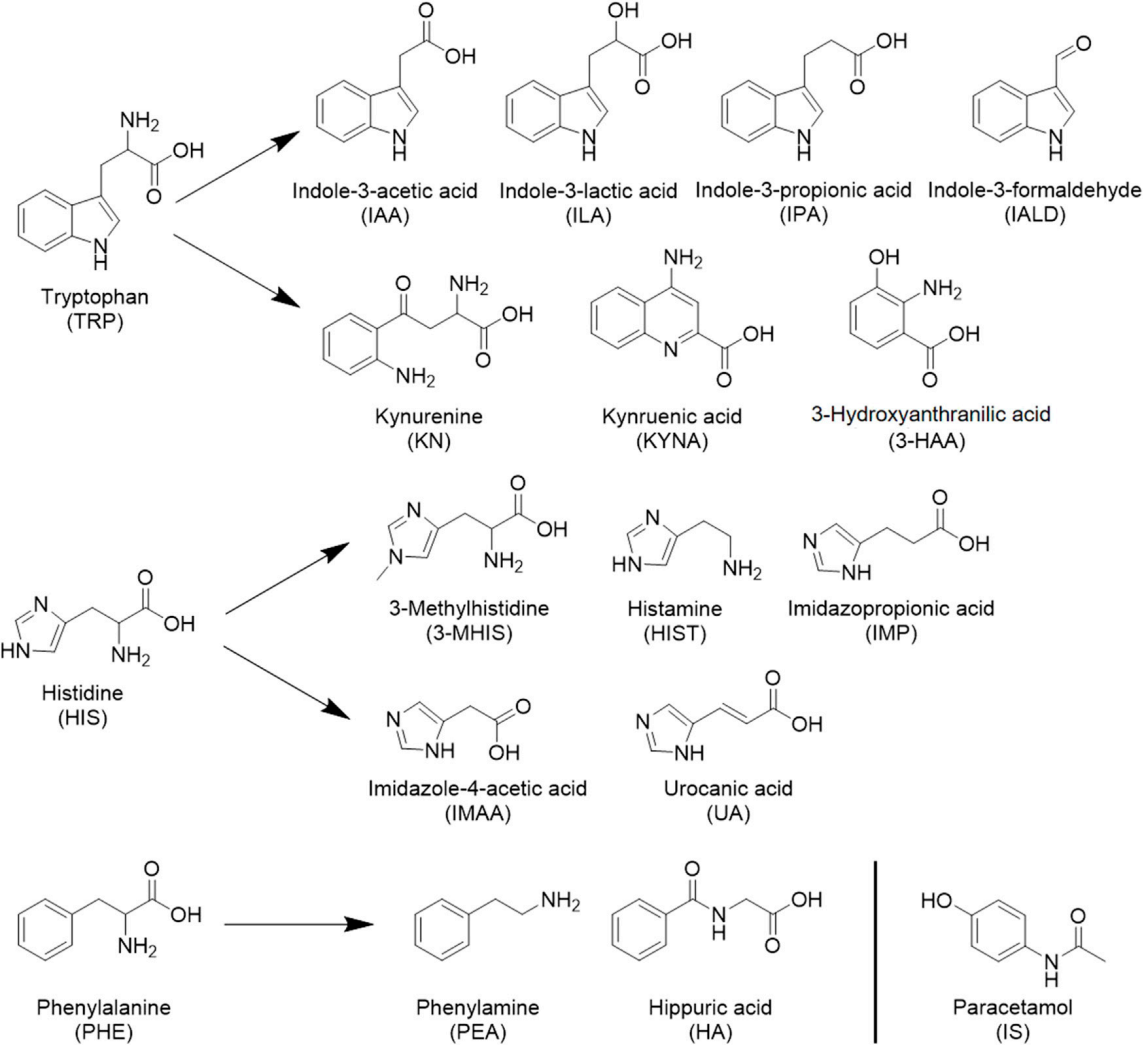
Seventeen metabolites and internal standard structural formulas measured.

Mass spectrometric detection was performed using multiple reaction monitoring (MRM) with electrospray ionization (ESI). The structural formulas of the 17 metabolites and the internal standard determined in this paper are shown in Figure 1; all contain nitrogen groups, and the response is higher in positive ion mode. The optimized mass spectrometry parameters of the 17 metabolites—such as molecular weight, transition, and collision energy—are shown in Table 1.

#### Chromatographic conditions

In terms of column selection, the target compounds had almost no retention on Bridge C18 column (2.1 mm × 100 mm, 2.7 μm), while all compounds eluted within 0.5 min. On the Acquity UPLC HSS T3 column, the peak shape of the indole derivative exhibited severe tailing. Finally, a SVEA C18 opal column (250 mm × 4.6 mm × 5 μm) was selected, exhibiting good resolution and a symmetrical peak shape. The flow rate was 0.4 ml/min. Water with 0.1% formic acid (FA) was used as the aqueous mobile phase, and 0.1% FA in methanol: acetonitrile (1:1) was used as the organic mobile phase. When the organic phase is pure methanol or pure acetonitrile, the analyte exhibited a front or split peak phenomenon. When the organic phase was adjusted to methanol: acetonitrile (1:1), the peak shape of the analyte was more symmetrical. Considering that the mass spectrometry conditions were in positive ion mode and that most metabolites contain carboxyl groups, a certain concentration of acid was chosen for addition to the mobile phase to improve the mass spectral response and peak shape; 0.1% acetic acid, 0.2% acetic acid, 0.5% acetic acid, and 0.1 and 0.5% FA were successively tested. The addition of acetic acid delayed the metabolite peak and prolonged the time of the whole chromatographic method. The best response was obtained with 0.1% FA, so this was chosen for addition to the aqueous and organic phases. The gradient elution conditions were: 0–5 min, 30–95% B; 5–8 min, 95%; 8.01–16 min, 30% B. Considering the structural formulas of the 17 metabolites, it was difficult to obtain the isotopic internal standard related to each substance, so acetaminophen with a similar structure was selected.

#### Sample preparation

In the extraction of intestinal content, the extraction solvent and extraction method were mainly investigated. Water, methanol, and acetonitrile were considered when choosing the extraction solvent, with water extracting the best peak shape. The extraction methods considered the following: direct treatment after mixing, posttreatment with ice bath ultrasound for 1 h, and shaking at 150 rpm and 20°C for 1 h. There will be a small number of interference peaks with the same MRM after shaking or sonication. There was no significant difference in the response of the target compound compared with the direct treatment by mixing. Therefore, the samples were processed directly after mixing. In addition, the Trp metabolites measured in the literature are unstable under conventional processing conditions, and methanol containing 2% FA (Ma et al., 2021) or methanol containing 10 mg/ml ascorbic acid (Fuertig et al., 2016) is generally chosen as the precipitant. In this experiment, it was found that adding methanol containing 2% FA to the precipitating agent resulted in a better peak shape.

### Method validation

Method validation was performed to obtain repeatable, stable targeted metabolomics. To validate the endogenous compound analysis methods, the following three methods are mainly adopted: 1) use of an isotope-labeled internal standard; 2) adding a standard to a surrogate matrix (Virág et al., 2020); and 3) adding standards to an authentic matrix (Fuertig et al., 2016). In this experiment, the corresponding isotope-labeled internal standards of the 17 substances were difficult to obtain, and the endogenous substances in the matrix could not be completely removed by various treatment methods. Therefore, the standards were added to the authentic matrix for method validation.

#### Specificity and carryover

**FIGURE 2:**
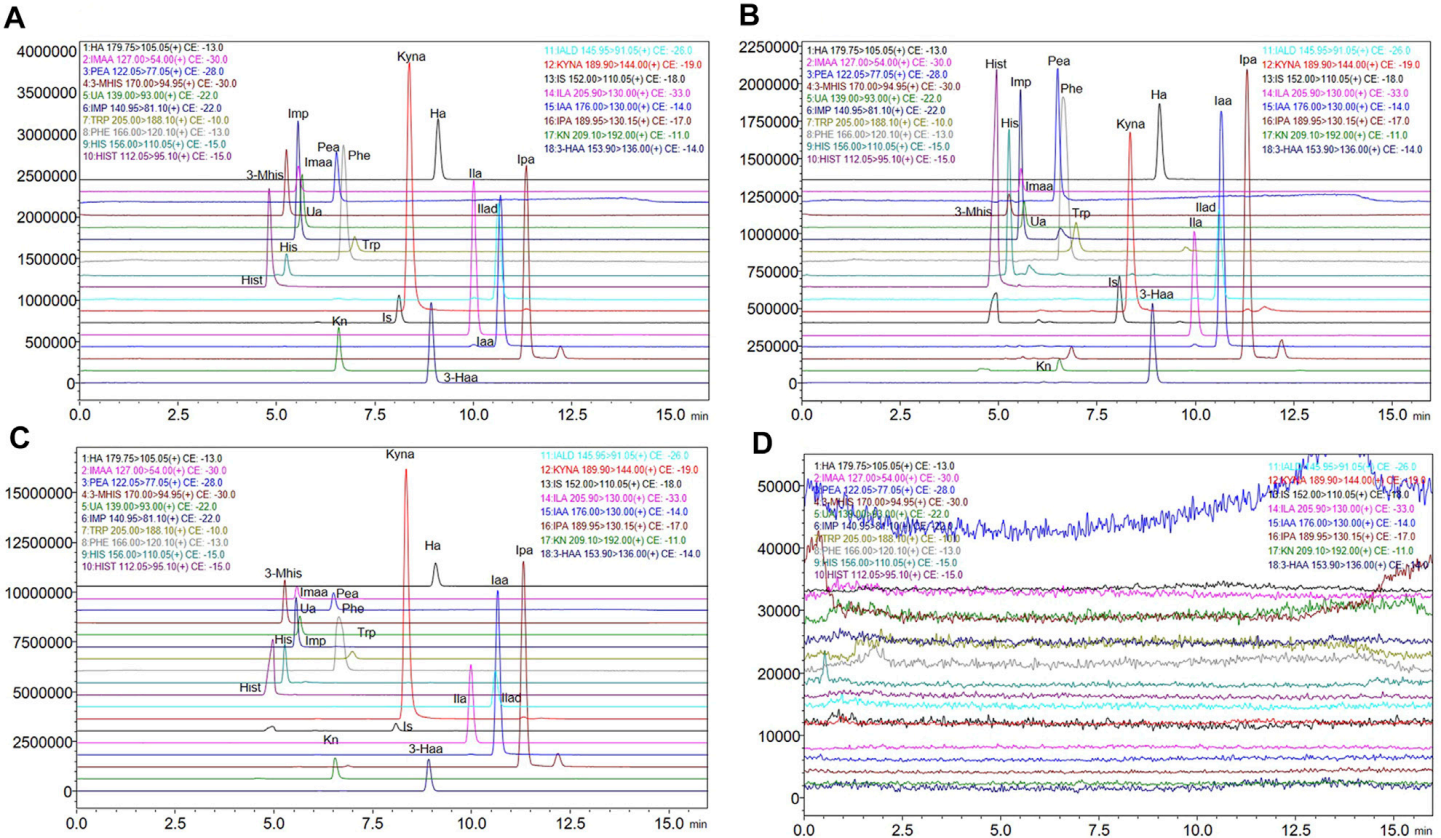
Extracted ion chromatograms of the 17 metabolites and internal standard. **(A)** Extracted ion chromatograms of standard solutions. **(B)** Extracted ion chromatograms of authentic matrix. **(C)** Extracted ion chromatograms of standard added into authentic matrix. **(D)** Extracted ion chromatograms of blank solution after five injections of the HQC standard).

Figures 2A–C shows the chromatograms of the standard substance, authentic matrix, and authentic matrix with standards. In the chromatogram of the authentic samples, all 17 metabolites corresponded to the retention time of the standard chromatogram. In authentic samples, multiple chromatographic peaks appear under the same MRM conditions, such as Imp, Trp, the IS and Ipa, but such compounds have proper chromatographic resolution. The peak shape of the chromatographic peaks after adding the standard to the authentic samples was good, and the retention time was consistent with that of the standard. After five consecutive high concentration QC (HQC) injections, the blank solvent chromatogram is shown in Figure 2D, indicating that there is no residue.

#### Linearity and lower limit of quantitation

Considering the concentration levels of these metabolites in intestinal bacterial samples, Trp, Phe, His, and imidazole propionic acid were basically at the microgram level, and the quantitative range was set as 100 ng/ml-12500 ng/ml. The quantitative range of other substances was set as 20 ng/ml-2500 ng/ml. A calibration curve for the analytes was built using the peak area ratio of each analyte to internal standard versus analyte concentration, plotted using 1/x weighted least squares linear regression. The correlation coefficient *R* ^2^ was used to represent the linearity, and the *R* ^2^ values of the 17 substances were all greater than 0.99. Since the metabolites in the matrix cannot be removed, we used the standard solution to calculate the lower limit of quantitation (LLOQ) and lower limit of detection (LLOD), as shown in Table 2.

**TABLE 2:** Retention time, linearity, quantitative range, and lower limit of quantitation of 17 metabolites.

Analyte	Rt (min)	Linear range(ng/ml)	*R* ^2^	LLODng/ml	LLOQng/ml
Ha	9.1	20–2500	0.996	1	2
Imaa	5.5	20–2500	0.999	0.5	2
Pea	6.5	20–2500	0.996	0.5	1
3-Mhis	5.2	20–2500	0.994	1	2
Ua	5.6	20–2500	0.991	1	2
Imp	5.5	100–12500	0.998	0.05	0.1
Trp	7.1	100–12500	0.997	0.5	1
Phe	8.7	100–12500	0.997	0.5	1
His	5.2	100–12500	0.999	0.1	1
Hist	4.8	20–2500	0.993	1	5
Iald	10.6	20–2500	0.999	0.5	2
Kyna	8.5	20–2500	0.997	1	2
Ila	10.1	20–2500	0.997	1	5
Iaa	10.7	20–2500	0.999	0.5	1
Ipa	11.3	20–2500	0.993	0.5	1
Kn	6.5	20–2500	0.993	0.5	1
3-Haa	8.9	20–2500	0.992	0.5	1

#### Accuracy and precision

According to the verification principle of the biological sample analysis method in the *Chinese Pharmacopoeia*, the low concentration QC (LQC), middle concentration QC (MQC), and high concentration QC (HQC) concentrations of Trp, Phe, His, and Imp were set as 200 ng/ml, 2000 ng/ml, and 10000 ng/ml, respectively. The LQC, MQC, and HQC concentrations of the other 13 metabolites were set to 40 ng/ml, 400 ng/ml, and 2000 ng/ml, respectively. The precision and accuracy of the 17 metabolites are shown in Table 3. The intraday accuracy was 88.80–107.50%, and the RSD was 1.72–11.53%. The inter-day accuracy was 90.38–114.01%, and the RSD was 2.96–10.78%, which were within the acceptable range, as shown in Table 3.

**TABLE 3:** Precision and accuracy of 17 metabolites.

Analyte	Concentrationng/ml	Intraday(*n* = 5)		Interday (*n* = 15)	
	LQC	Accuracy (%)	RSD (%)	Accuracy (%)	RSD (%)
	MQC				
	HQC				
Ha	40	94.97	11.53	96.27	7.01
	400	100.06	5.37	105.82	5.06
	2000	92.03	5.40	97.37	5.22
Imaa	40	100.88	4.99	101.32	5.33
	400	104.59	4.57	106.80	3.55
	2000	95.00	2.11	97.74	4.30
Pea	40	92.98	3.50	97.79	5.85
	400	101.14	4.86	106.14	4.57
	2000	102.98	5.88	99.03	6.70
3-Mhis	40	95.05	10.21	97.35	6.57
	400	102.40	3.99	104.51	3.80
	2000	92.33	3.23	96.71	5.16
Ua	40	100.84	5.44	100.80	5.32
	400	104.19	3.96	106.81	3.35
	2000	91.99	2.28	95.68	4.39
Imp	200	92.16	8.25	94.94	6.70
	2000	107.50	3.79	114.01	7.28
	10000	92.07	2.92	95.01	6.42
Hist	40	95.69	6.15	98.64	5.13
	400	103.14	5.80	103.32	5.42
	2000	92.20	2.84	93.53	3.05
Iald	40	94.02	3.95	95.51	3.74
	400	102.19	4.56	104.95	3.41
	2000	92.34	2.03	97.13	4.65
Kyna	40	96.11	8.39	97.60	5.94
	400	102.98	3.61	103.89	3.09
	2000	93.18	3.95	98.90	5.78
Ila	40	98.64	4.78	100.32	3.49
	400	95.52	4.53	102.78	6.56
	2000	92.92	3.79	96.03	5.50
Iaa	40	99.68	5.11	97.45	4.09
	400	98.38	3.60	103.71	5.03
	2000	101.64	3.18	99.38	3.15
Ipa	40	97.71	5.80	98.63	4.28
	400	100.82	3.34	104.81	3.96
	2000	90.69	1.72	96.09	5.14
Kn	40	100.11	1.98	99.32	3.38
	400	98.31	4.41	103.59	4.90
	2000	89.45	2.66	95.88	8.37
3-Haa	40	98.88	3.12	100.65	3.24
	400	98.15	2.42	103.25	5.64
	2000	94.51	2.15	98.32	5.15
Trp	200	89.30	5.98	95.23	7.51
	2000	98.02	1.74	106.22	6.79
	10000	96.71	5.16	99.75	4.47
Phe	200	88.80	7.14	93.79	8.41
	2000	99.01	3.08	107.58	6.86
	10000	91.40	3.13	96.52	5.90
His	200	98.54	7.76	90.38	10.78
	2000	104.81	4.10	107.71	5.37
	10000	94.44	3.19	96.20	2.96

#### Stability

Taking the needs of practical experiments into account, the spiked samples were placed at 4°C for 12 h before or after treatment or freeze-thawed at −20°C thrice before treatment. The stability under the three conditions is shown in Table 4, and all metabolites are in the range of 85–115%, which meets the requirements of biological sample analysis.

**TABLE 4:** Stability of 17 metabolites under three conditions.

Analyte	Concentration (ng/ml)	Stability before treatment (4°C 12 h)		Stability after treatment (4°C 12 h)		Repeated freezing and thawing 3 times (-20°C)	
	LQC	Mean (%)	SD (%)	Mean (%)	SD (%)	Mean (%)	SD (%)
	MQC						
	HQC						
Ha	40	103.12	7.11	95.10	6.29	91.00	5.77
	400	103.14	6.28	92.98	5.01	100.42	3.87
	2000	105.99	6.12	93.86	3.11	99.08	2.36
Imaa	40	98.58	4.16	90.81	6.18	98.56	5.89
	400	97.64	5.05	94.79	2.81	104.90	3.96
	2000	101.76	3.59	92.29	4.26	96.13	1.67
Pea	40	107.75	2.88	92.29	4.36	91.77	6.07
	400	101.63	4.12	95.94	4.84	109.92	4.50
	2000	92.65	7.57	99.67	4.27	97.11	2.01
3-Mhis	40	107.05	11.84	94.43	5.73	96.83	5.95
	400	97.94	2.29	93.84	3.36	104.44	4.20
	2000	102.41	3.75	92.62	4.18	92.59	3.41
Ua	40	100.10	5.50	98.73	3.05	91.14	6.18
	400	102.25	3.80	95.06	4.22	106.14	2.29
	2000	102.59	3.16	97.82	2.65	96.98	3.38
Imp	200	110.04	10.35	103.03	6.33	92.72	8.68
	2000	99.69	4.17	85.06	5.66	98.00	1.83
	10000	99.94	3.52	96.56	4.75	93.18	2.03
Hist	40	102.12	7.19	97.87	9.26	90.21	2.32
	400	103.69	4.84	101.13	6.69	88.34	8.83
	2000	97.30	4.72	100.27	6.47	93.78	1.91
Iald	40	107.84	3.48	105.49	4.76	91.34	4.35
	400	104.79	2.72	88.89	3.73	107.27	3.37
	2000	106.78	2.02	92.16	3.96	98.43	1.69
Kyna	40	103.97	7.14	98.91	6.38	99.91	5.57
	400	104.08	2.52	98.12	4.24	107.81	3.96
	2000	109.31	4.15	94.71	2.74	91.24	3.02
Ila	40	102.94	4.12	98.54	1.34	92.41	7.63
	400	108.75	5.87	95.84	2.93	99.43	5.21
	2000	101.77	3.98	93.89	3.67	93.59	1.99
Iaa	40	100.49	5.05	102.97	4.99	90.68	2.77
	400	109.14	1.61	102.67	6.02	102.22	4.59
	2000	96.13	3.62	100.45	2.33	97.10	3.37
Ipa	40	102.44	9.15	95.92	3.95	91.87	4.24
	400	98.62	4.78	89.72	3.19	110.39	1.76
	2000	106.94	3.78	94.39	2.90	95.49	1.23
Kn	40	98.20	3.18	98.79	8.43	89.68	3.87
	400	96.98	5.06	88.73	2.85	104.88	4.98
	2000	100.10	4.67	95.39	3.95	90.56	1.87
3-Haa	40	100.25	6.17	95.97	3.96	88.63	9.39
	400	101.65	3.82	96.55	5.20	100.50	3.51
	2000	101.87	2.51	88.61	2.45	95.02	3.30
Trp	200	101.89	4.95	89.90	6.94	89.65	7.47
	2000	106.21	2.75	106.35	3.10	99.13	3.44
	10000	98.02	6.60	105.63	4.43	95.38	1.29
Phe	200	102.35	6.75	91.91	7.36	88.96	3.32
	2000	109.05	3.78	101.04	3.16	97.09	3.89
	10000	106.54	3.50	102.75	2.49	96.36	1.86
His	200	94.62	9.56	107.88	8.31	95.42	4.21
	2000	101.91	4.50	105.82	4.53	98.44	5.37
	10000	104.70	3.23	100.81	4.44	100.94	1.49

#### Extraction recovery

The extraction recovery rate = the concentration of the sample after extraction/the concentration of the sample before extraction * 100%; the extraction recovery rate of 17 substances was 88.34–114.26%, which met the requirements of biological sample analysis (see Table 5.

**TABLE 5:** Extraction recoveries of 17 metabolites.

Analyte	Concentration (ng/ml)	Extraction recovery	
	LQC	Mean (%)	SD (%)
	MQC		
	HQC		
Ha	40	97.68	6.80
	400	104.24	3.30
	2000	99.76	3.83
Imaa	40	101.16	5.58
	400	105.07	2.11
	2000	107.34	1.70
Pea	40	104.89	4.08
	400	104.50	1.43
	2000	103.99	1.68
3-Mhis	40	100.22	6.69
	400	101.41	3.44
	2000	105.77	3.43
Ua	40	99.39	5.30
	400	102.54	4.99
	2000	107.48	2.77
Imp	200	104.15	2.48
	2000	104.36	2.05
	10000	113.49	3.19
Hist	40	99.99	5.10
	400	101.02	3.24
	2000	103.76	5.89
Iald	40	109.19	6.13
	400	102.36	1.32
	2000	111.08	5.23
Kyna	40	98.77	3.18
	400	102.92	3.96
	2000	104.35	3.80
Ila	40	101.59	6.16
	400	105.04	2.42
	2000	105.62	1.95
Iaa	40	101.73	3.31
	400	100.37	2.26
	2000	91.47	3.10
Ipa	40	99.57	2.66
	400	103.04	1.14
	2000	114.26	5.34
Kn	40	105.06	4.72
	400	101.19	1.95
	2000	110.80	2.85
3-Haa	40	98.28	5.04
	400	99.73	4.21
	2000	106.02	2.86
Trp	200	96.81	4.03
	2000	96.05	3.32
	10000	96.61	1.64
Phe	200	108.99	7.94
	2000	103.38	1.51
	10000	101.32	2.83
His	200	98.65	8.08
	2000	91.23	1.88
	10000	88.34	3.54

#### Matrix effect

The results showed that the matrix effect was consistent at different levels and in the range of 86.38–112.64%, as shown in Table 6.

**TABLE 6:** Matrix effects of 17 metabolites.

Analyte	Concentration (ng/ml)	Matrix effect	
	LQC	Mean (%)	SD (%)
	MQC		
	HQC		
Ha	40	95.08	9.82
	400	91.14	4.82
	2000	91.54	3.46
Imaa	40	98.59	7.81
	400	98.17	3.19
	2000	99.36	3.72
Pea	40	93.12	2.19
	400	93.67	4.13
	2000	112.64	9.19
3-Mhis	40	96.16	12.38
	400	97.58	1.77
	2000	96.64	1.94
Ua	40	102.04	4.19
	400	96.28	2.13
	2000	97.23	2.32
Imp	200	97.18	12.70
	2000	87.59	3.91
	10000	101.66	5.05
Hist	40	96.69	4.60
	400	104.55	3.60
	2000	99.66	2.66
Iald	40	95.95	5.28
	400	96.28	2.95
	2000	93.89	2.83
Kyna	40	95.90	9.42
	400	97.77	2.80
	2000	94.84	3.66
Ila	40	97.98	5.59
	400	91.93	4.89
	2000	99.88	3.27
Iaa	40	104.74	5.92
	400	93.62	4.70
	2000	103.61	2.70
Ipa	40	99.15	7.27
	400	93.05	3.20
	2000	94.62	1.51
Kn	40	101.29	3.92
	400	91.96	3.52
	2000	97.16	1.09
3-Haa	40	96.66	5.33
	400	96.06	5.90
	2000	98.09	3.36
Trp	200	89.21	5.15
	2000	92.39	3.32
	10000	97.68	4.79
Phe	200	86.38	6.97
	2000	90.47	2.36
	10000	96.06	2.53
His	200	106.30	10.33
	2000	98.39	3.04
	10000	98.16	1.87

### Establishment of the rheumatoid arthritis rat model

**FIGURE 3:**
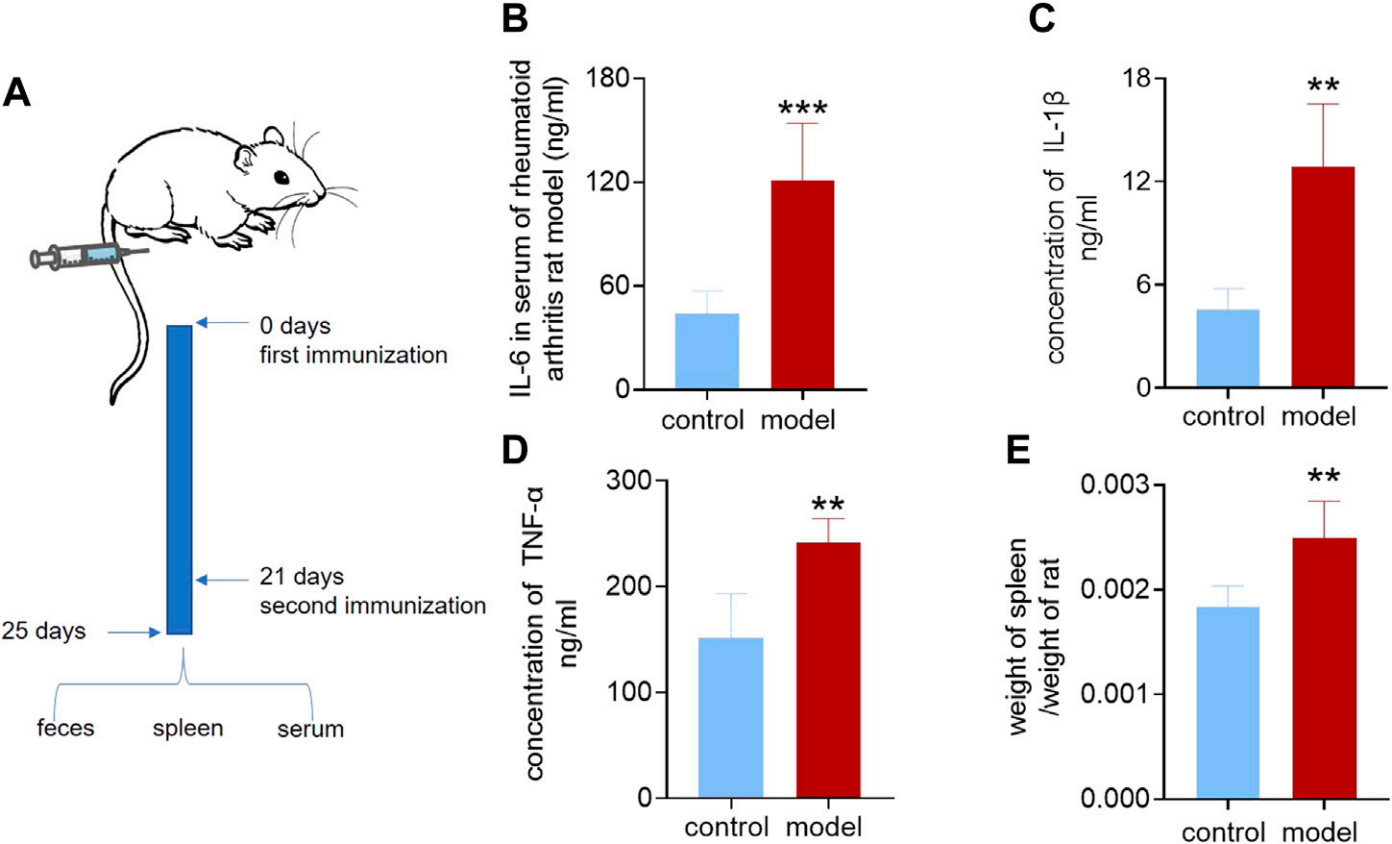
Inflammatory factors and immunization index of the rheumatoid arthritis rat model. **(A)** Establishment of the rheumatoid arthritis rat model. **(B)** Interleukin-6 in serum of the rheumatoid arthritis rat model and control group. **(C)** Interleukin-1β in serum of rheumatoid arthritis rat model and control group. **(D)** Tumor necrosis factor-α in serum of the rheumatoid arthritis rat model and control group. **(E)** Ratio of spleen weight to body weight of rheumatoid arthritis rat model and control group. A two-sided *t* test was used, and *p* < 0.05 was considered statistically significant. **p < 0.01, ***p < 0.001.

We applied the previously established targeted omics approach to the model of rheumatoid arthritis, a classic inflammation-related disease. We first demonstrated the successful establishment of rheumatoid arthritis. The model group was initiated by intradermal injection of complete Freund’s adjuvant (CFA) at the base of the hind paw region (Choudhary et al., 2018) and recorded as Day 0. On the second day, the injected soles of the rats were observed to be red and swollen. On the 21st day, when the rats were injected again, the toe and ankle joints were observed to be swollen. The other soles that were not injected also showed redness and swelling of the soles and joints. On the 25th day, the rats were weighed, blood and feces were collected, and the gut microbiota, feces, and spleen were collected after sacrifice. The process is shown in Figure 3A. Using a kit to measure inflammatory factors in plasma, it was found that, compared with the control group, the inflammatory factors IL-6, IL-1β, and TNF-α were significantly increased in the model group, as shown in Figures 3B–D. The spleen and rats were weighed, and the spleen weight/body weight was recorded as the immune index. The immune index in the model group was significantly increased, as shown in Figure 3E, which proved that the model was successfully constructed.

### 16S RNA sequencing of feces of rheumatoid arthritis rats and determination of the intestinal contents

**FIGURE 4:**
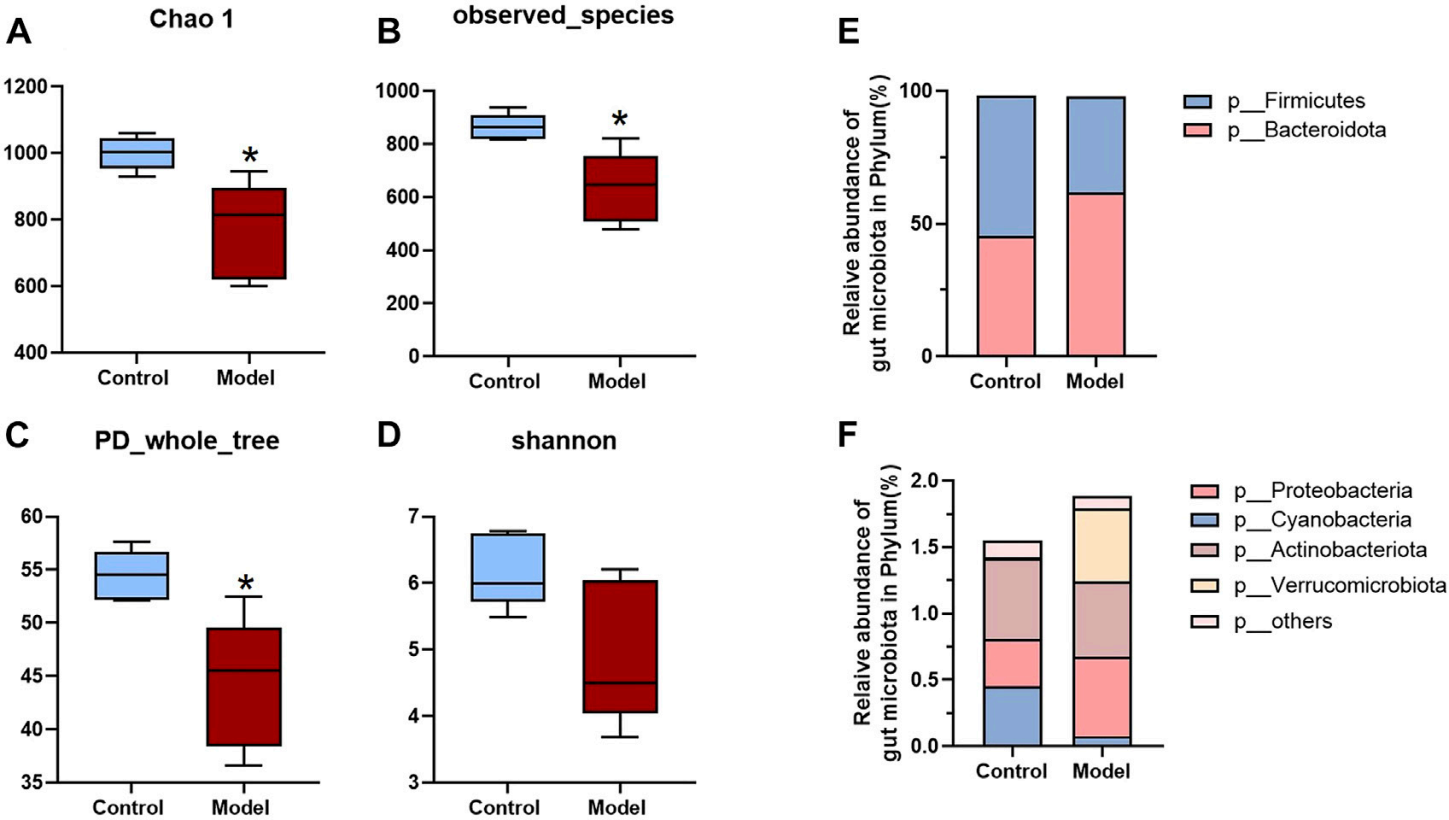
Analysis of fecal biodiversity and phylum level of RA rats. **(A–D)** Chao 1, Observed_ species, PD_ whole_ trees and Shannon indices of model and control groups. **(E,F)** The relative abundance of bacteria at the phylum level. A two-sided *t* test was used, and *p* < 0.05 was considered statistically significant. *p < 0.05, **p < 0.01.

**FIGURE 5:**
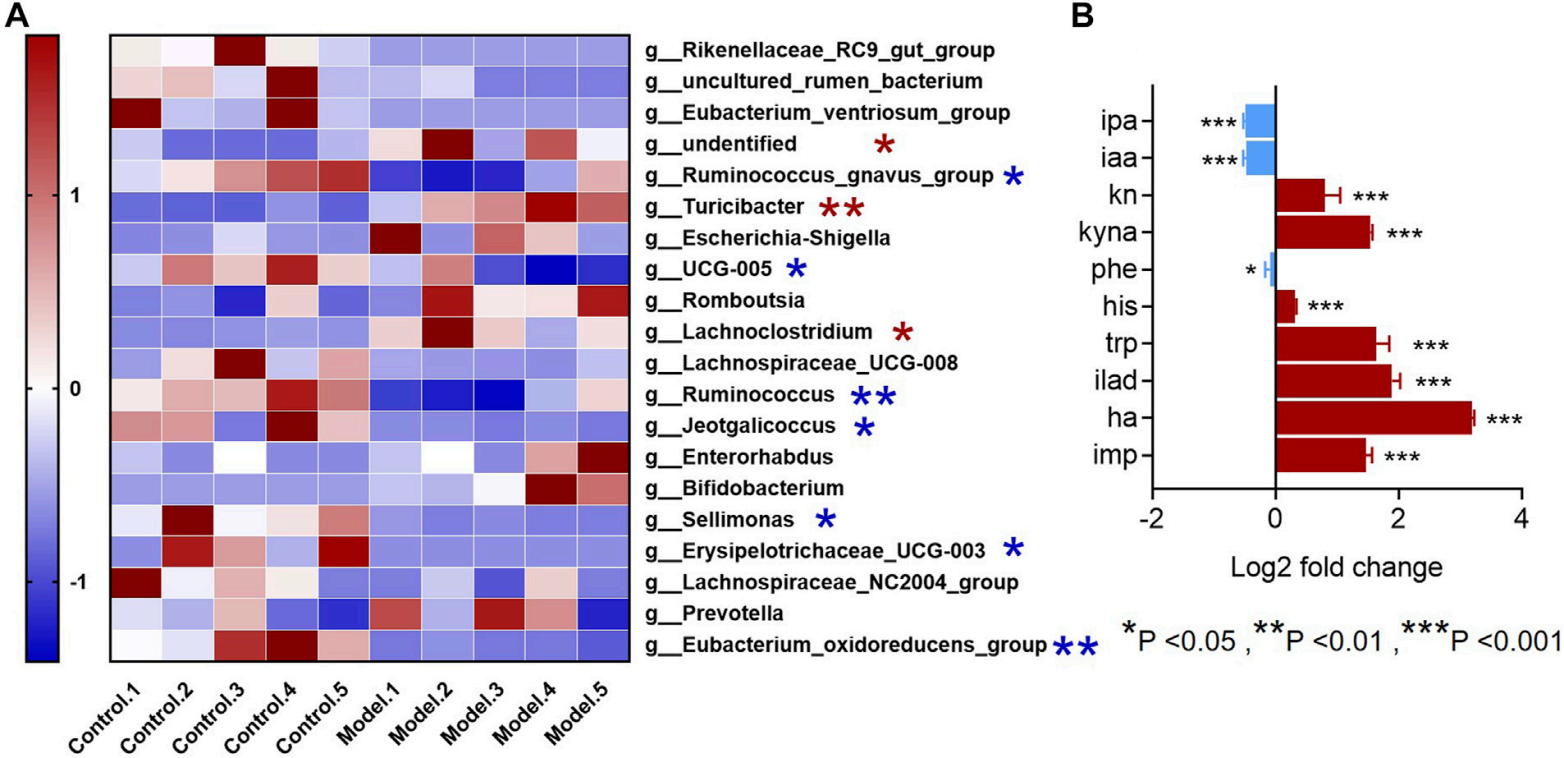
Genus-level differences in fecal microbiota and metabolite differences in intestinal microbiota samples in the RA model. **(A)** The top-20 bacterial genera with the most substantial change in abundance after the establishment of rheumatoid arthritis rat model. (*The red pentagram represents bacteria with abundance increased after the establishment of rheumatoid arthritis model. *The blue pentagram represents bacteria with decreased abundance after the establishment of rheumatoid arthritis model.) **(B)** Differences in metabolites in the gut microbiota of model rats and the control group. A two-sided *t* test was used and *p* < 0.05 was considered statistically significant. *p < 0.05, **p < 0.01, ***p < 0.001.

Since all the metabolites we measured were microbiota metabolites, we also examined the changes of intestinal microbiota in rats with rheumatoid arthritis. We hoped to determine whether there is a correlation between changes in intestinal microbiota and changes in these metabolites in rats with rheumatoid arthritis. The feces of model and control rats were analyzed by 16S sequencing. Compared with the control group, the *α*-biodiversity of the model group decreased, as shown in Figures 4A–D. Analysis of the gut microbiota in the feces revealed dysbiosis. As shown in Figures 4E,F, the phylum level is mainly composed of Firmicutes and Bacteroidetes, accounting for more than 95% of the total bacteria—similar to the composition of the human gut microbiota (Turnbaugh et al., 2006). The abundance of Bacteroidetes in the model group increased while the abundance of Firmicutes was decreased. In other phyla with relatively low contents, the abundance of Proteobacteria and Verrucomicrobiota was elevated and the abundance of Cyanobacteria decreased. As shown in Figure 5A, at the genus level, the relative abundances of *g__undentified*, *g__Turicibacter*, and *g__Lachnoclostridium* increased and the relative abundances of *g__Ruminococcus_gnavus_group*, *g__UCG-005*, *g__Ruminococcus*, *g__Jeotgalicoccus*, *g__Sellimonas*, *g__Erysipelotrichaceae_UCG-003*, and *g__Eubacterium_oxidoreducens_group* decreased compared to the control group. The intestinal contents samples were analyzed, as shown in Figure 5B. Compared with the control group, the three amino acids in the model group changed significantly: Phe was significantly decreased (*p* < 0.05), and His and Trp were significantly increased (*p* < 0.001). In the Trp pathway, Iaa and Ipa were significantly decreased (*p* < 0.001 and *p* < 0.001), and Iald, Kn, and Kyna were significantly increased (*p* < 0.001, *p* < 0.001, and *p* < 0.001). In the Phe pathway, Ha was significantly elevated (*p* < 0.001). In the His pathway, Imp was significantly elevated (*p* < 0.001).

### Screening of drugs for RA *in vitro*

The field of gut bacteria is a new direction in studying the mechanism of drug action. Here, we selected several Chinese patent drugs and their effective components commonly used in the clinical treatment of arthritis and used the targeted omics method established by us to provide new ideas for the study of their mechanism of action from the perspective of intestinal bacteria. *Tripterygium wilfordii* and *Paeonia lactiflora* are both commonly used Chinese herbal medicines for the treatment of rheumatoid arthritis (Zhang and Wei, 2020; Zhang Y. et al., 2021). We chose total glucosides of paeony (the extract composition of white paeony capsules is shown in Supplementary Table S1). Their active ingredients are paeoniflorin, albiflorin std, and benzoylpaeoniflorin. *Tripterygium wilfordii* polyglycoside tablets (the extract composition of which is shown in Supplementary Table S2) has as active ingredients triptolide, celastrol, wilforine, wilforlide A, and triptonide. We incubated these drugs *in vitro* with the gut bacteria of SD rats and then measured them with established targeted metabolomics for a preliminarily exploration of whether they can change the concentration of these metabolites.

**FIGURE 6:**
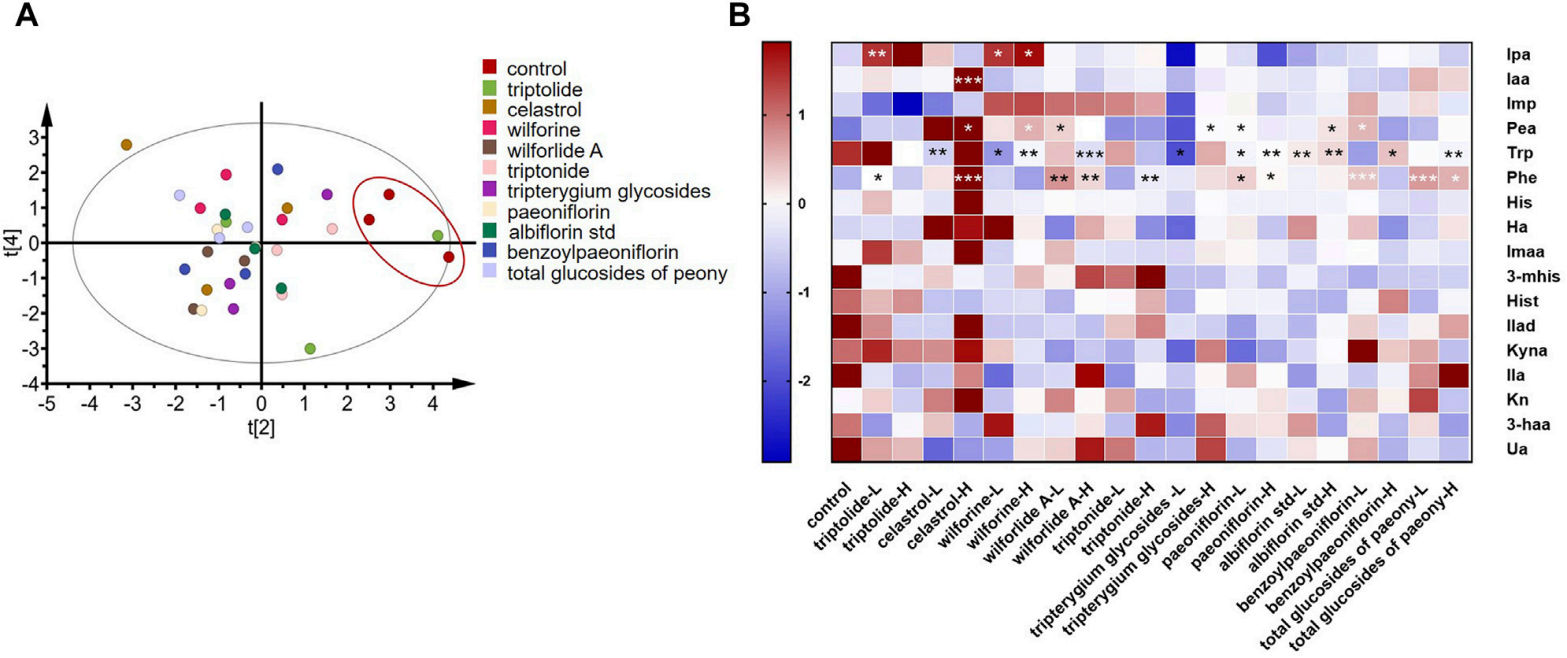
Screening of drugs for rheumatoid arthritis *in vitro*. **(A)** Principal component analysis plots of *in vitro* high-dose group samples. **(B)** Changes in metabolites after drug treatment *in vitro*. A two-sided *t* test was used and *p* < 0.05 was considered statistically significant. *p < 0.05, **p < 0.01, ***p < 0.001. (L means low dose 10 ug/ml, H means high dose 100 ug/ml, “*Tripterygium* glycoside” represents *Tripterygium wilfordii* polyglycoside tablets, “total glucosides of peony” represents total glucosides of white paeony capsules.)

As shown in Figure 6A, principal component analysis (PCA) was performed on 11 high-dose groups and a control group. It was found that, except for the triptolide group, the other groups were separated from the control group. These results showed that the addition of drugs changed metabolites in the *in vitro* incubation system compared to the control group. As shown in Figure 6B, *Tripterygium* glycoside and its active ingredient groups, the high-dose triptolide group, the low-dose wilforine group, and the high-dose wilforine group showed a significant increase of indole propionic acid in gut bacteria (*p* < 0.01, *p* < 0.05, *p* < 0.05). The high-dose celastrol group showed a significant increase of indoleacetic acid (*p* < 0.001). The high-dose celastrol group, high-dose wilforine group, low-dose wilforlide A group, and high-dose *Tripterygium* glycoside group showed significant increase of Pea (*p* < 0.05, *p* < 0.05, *p* < 0.05, and *p* < 0.05). The low-dose celastrol group, the low-dose and high-dose wilforine groups, the high-dose wilforlide A group, and the low-dose *Tripterygium* glycoside group showed a significant decrease of Trp (*p* < 0.01, *p* < 0.05, *p* < 0.01, *p* < 0.001, *p* < 0.05). The low-dose triptolide group, the high-dose celastrol group, the low-dose and high-dose wilforlide A groups, and the high-dose triptonide group significantly elevated Phe (*p* < 0.05, *p* < 0.001, *p* < 0.01, *p* < 0.01, and *p* < 0.01). In the total glucosides of paeony and its active components, the low-dose paeoniflorin group, the high-dose albiflorin std group, and the low-dose benzoyl paeoniflorin group significantly elevated Pea (*p* < 0.05, *p* < 0.05, *p* < 0.05). The low-dose and high-dose paeoniflorin groups, the low-dose and high-dose albiflorin std groups, the high-dose benzoyl paeoniflorin group, and the high-dose total glucosides of paeony group can significantly decrease Trp (*p* < 0.05, *p* < 0.01, *p* < 0.01, *p* < 0.01, *p* < 0.05, and *p* < 0.01). The low-dose and high-dose paeoniflorin group, low-dose benzoyl paeoniflorin group, and low-dose and high-dose total glucosides of paeony group can significantly elevate Phe (*p* < 0.05, *p* < 0.05, *p* < 0.001, *p* < 0.001, and *p* < 0.05) (*p* values of differential metabolites compared with the control group after incubation with drugs or compounds are shown in Supplementary Table S3). Therefore, gut microbiota derived metabolites in Trp and Phe pathways occurred in variations after incubation with *Tripterygium* glycosides and their active components, including two increasing beneficial Trp metabolites: indole propionic acid and indole acetic acid. Concentrations of phenylethylamine in the gut microbiota increased after incubation with total glucosides and active components of peony.

## Discussion

We successfully developed a simple, rapid, and derivatization-free LC–MS/MS method for the simultaneous determination of 17 metabolites targeting Phe, Trp, and His in intestinal content, targeting three metabolic pathways. The method has good specificity, and high sensitivity, accuracy, precision, and recovery rate, meets the requirements of biological sample analysis, and can be successfully applied to bacterial samples.

In this experiment, a rat model of rheumatoid arthritis was selected, and dysbiosis was found in the feces of the model group. In our experiments, increased abundances of *g__Turicibacter* and *g__Lachnoclostridium* and decreased abundances of *g__Erysipelotrichaceae_UCG-003* were observed in the model group. *g__Turicibacter* belongs to the phylum Firmicutes (Maki and Looft, 2022) and is a strictly anaerobic Gram-positive rod-shaped bacterium (Bosshard et al., 2002), the specific function of which is still unclear. However, studies have shown that it is positively correlated with a variety of inflammatory diseases and its abundance is increased in hepatitis mice (Yang et al., 2020a; Somm et al., 2021). One study showed that the use of icariin significantly reduced the abundance of the genus *g__Turicibacter* in colitis mice (Zhang H. et al., 2021). Similarly, the same phenomenon occurred during the treatment of colitis mice with caffeic acid (Wan et al., 2021). Its abundance was significantly increased in a chronic inflammation-based mouse model of atherosclerosis and was significantly positively correlated with plaque area in the mouse aorta (Huang K. et al., 2021). *g__Lachnoclostridium* produces short-chain fatty acids mainly through the 4-aminobutyrate/succinate pathway (Zhao E. et al., 2021) and is a proinflammatory bacterium. Its relative abundance is significantly increased in mice with ulcerative colitis (Wang et al., 2018), patients with eosinophilic inflammation (Kim et al., 2020), and mice with atherosclerosis (Sun et al., 2021). *g__Erysipelotrichaceae_UCG-003* is a butyric acid-producing bacterium that can be induced by helper T-cell 17 (Th17 cells) (Cheng et al., 2021); a clinical experiment showed that the bacterium was more abundant in healthy aging volunteers than in the diseased aging group (Singh H. et al., 2019). Another clinical study showed that the abundance of this bacterium was significantly decreased in lung cancer patients compared to healthy patients (Zhao F. et al., 2021). Therefore, it is speculated that *g__Erysipelotrichaceae_UCG-003* is a beneficial bacterium, although the specific mechanism is still unclear. Less consistent with the literature is the decreased abundance of *g__Ruminococcus_gnavus_group* in the model group. This is a mucin-degrading gut bacterium (Ahn et al., 2022) belonging to the phylum Firmicutes (Graziani et al., 2016) and is enriched in patients with inflammatory bowel disease. *g__Ruminococcus_gnavus_group* can produce an inflammatory polysaccharide, which can induce dendritic cells to produce inflammatory cytokines such as TNF-α, further leading to the progression of Crohn’s disease. Although not the same as our research results, a study based on genomic analysis of *g__Ruminococcus_gnavus_group* mainly secreted glycoside hydrolase and polysaccharide lyase, suggesting that these bacteria may be closer to the intestinal mucosa or adhere to the position of the intestinal mucosa (Graziani et al., 2016). Therefore, this bacterium has a greater effect on inflammatory bowel disease, but the mechanism may be different from that of rheumatoid arthritis. The functions of *g__undentified*, *g__Eubacterium_oxidoreducens_group*, *g__Jeotgalicoccus*, and *g__Sellimonas* are not very well studied, this being the first time they have been found to be significantly reduced in rheumatoid arthritis rats. Unfortunately, in the rat model of rheumatoid arthritis, the different genera we found were not related to the metabolism of the three amino acids we measured. Therefore, we did not find a correlation between changes in gut bacteria and changes in these metabolites in rats with rheumatoid arthritis—one of the limitations of our study. However, many of these different bacteria were related to butyric acid metabolism, so our results suggest that butyric acid can also be a direction for research on rheumatoid arthritis and intestinal bacteria which is worthy of further exploration.

Furthermore, we used the established targeted metabolomics method to measure the concentration of the metabolites in intestinal content of the model mice; differential metabolites were found. Iaa and Ipa were significantly decreased, but Iald, Kn, Kyna, Ha, and Imp were significantly increased. Among them, Iaa and Ipa were negatively correlated with inflammation. These indole derivatives can act on pregnane X receptors (PXR) and AhR and have a certain inhibitory effect on inflammatory diseases such as colitis (Huang W. et al., 2021), arthritis (Rosser et al., 2020), steatohepatitis (Ji et al., 2019; Zhao et al., 2019), ankylosing spondylitis (Shen et al., 2022), and obesity (Su et al., 2022)—consistent with our experimental results. Trp is metabolized by bacteria into two pathways, indole derivatives and kynurenine, which antagonize each other. Therefore, the change trend of Kn and Kyna is opposite to that of indole derivatives. Studies have shown that Iald induces IL-22 by activating the AHR pathway and has an inhibitory effect on inflammation (Teng et al., 2018). However, another study showed that, although Iald has anti-inflammatory activity *in vitro*, it has pro-osteoclastogenesis and pro-angiogenic effects (Langan et al., 2021), which may be the reason for the significantly higher concentration of Iald in the model group. Hippuric acid, a biomarker identified from the urine of rheumatoid arthritis rats, inhibits osteoclast production *in vitro* to prevent osteoclasts from increasing bone resorption; this may be related to the underlying mechanism of rheumatoid arthritis (Jiang et al., 2016; Zhao et al., 2020). Ha has been identified as a biomarker of rheumatoid arthritis, and a study has revealed that hippuric acid inhibits osteoclast production *in vitro* to prevent osteoclasts from increasing bone resorption; this may be related to rheumatoid arthritis. Imidazole propionic acid is a newly discovered product of His metabolism by intestinal bacteria that positively correlates with systemic inflammation and is a biomarker related to diabetes (Koh et al., 2018). One study showed that it was negatively correlated with anti-inflammatory bacteria (Molinaro et al., 2020), which is consistent with our experimental results. In conclusion, these metabolites of intestinal content in rheumatoid arthritis model rats appeared varied. We then screened *Tripterygium* glycosides, total peony glucosides, and their active ingredients *in vitro*. The results showed that *Tripterygium* glycosides could modulate Trp and Phe pathways, especially Ipa, Iaa, and Pea. Total peony glucosides can modulate the Phe pathway, especially phenylethylamine. It has been documented in the literature that phenethylamine has an effect on the progression of chronic inflammation in humans (Maráková et al., 2020), especially neuroinflammation (Chen et al., 2022). However, phenethylamine has been less studied in nonbrain inflammatory diseases.

There is currently no targeted metabolomic method of simultaneously targeting the inflammatory markers of these three amino acid pathways. Due to the close connection between inflammatory diseases and intestinal microbiota, it is very important to establish our method. However, our experiment still has certain limitations. Some different genera were identified in the analysis of the fecal samples of the model rats but the functional analysis did not find that these genera had metabolic effects on the three amino acid pathways. Therefore, further *in vivo* experiments may be required for confirmation. In conclusion, it was found that the pathogenesis of RA was related to dysbacteriosis, that Iaa and Ipa in the bacterial microbiota were significantly decreased, and that Iald, Kn, Kyna, Ha, and Imp were significantly increased. Using the method of *in vitro* incubation, the commonly used drugs for the treatment of RA—*Tripterygium* glycosides, total peony glucosides, and their corresponding monomers —were screened, and metabolites in Trp and Phe pathways occurred. Their variations of Ipa, Iaa, and Pea deserve special attention, which may be related to the mechanism of the treatment of rheumatoid arthritis. Our method can identify targets for the interaction of inflammatory diseases and microbiota and can be applied to the mechanistic study and drug screening of other inflammatory diseases. However, our research existed some limitations in the *in vitro* screening of drugs. We selected the gut microbiome of healthy rats to incubate with drugs, which were more related to the drugs’ preventive effects. Further studies are needed to more widely apply our approach to drug screening for the treatment of RA and mechanism elucidation, including whether drugs can reverse gut microbiota-derived metabolites of RA model rats *in vitro*, whether drugs can reverse metabolites of gut microbiome in RA model rats after dosing of drugs, and whether drugs can reverse these metabolites in patients with rheumatoid arthritis. These are all worthy of further exploration.

## Conclusion

In summary, we established targeted metabolomics of bacterial inflammatory markers and completed method validation. A typical inflammatory disease model of rheumatoid arthritis was then established, and we successfully applied targeted metabolomics of inflammatory markers to this model of inflammatory disease. In addition, we screened rheumatoid arthritis drugs *in vitro* and found that their treatment of rheumatoid arthritis may be related to microbial metabolites. In conclusion, our study may provide new insights into the mechanisms of inflammatory diseases and drug screening.

## Data Availability

The data presented in the study are deposited in the NCBI database, accession number PRJNA877047.
