# Supplementary material for: Gut microbiota-derived metabolites in inflammatory diseases based on targeted metabolomics
Source: Front Pharmacol. 2022 Sep 27;13:919181. doi: 10.3389/fphar.2022.919181 (PMC9551995; doi:10.3389/fphar.2022.919181)
Supplement: Supplementary file 1 [file DataSheet1.docx]

Gut microbiota derived metabolites in inflammatory diseases based on targeted metabolomics

Hui Xu^1^, Li-Bin Pan^1^, Hang Yu^1^, Pei Han^1^, Jie Fu^1^, Zheng-Wei Zhang^1^, Jia-Chun Hu^1^, Xin-Yu Yang^1^, Adili Keranmu^1^, Hao-Jian Zhang^1^, Meng-Meng Bu^1^, Jian-Dong Jiang^1,^*, Yan Wang^1,^*

1. State Key Laboratory of Bioactive Substance and Function of Natural Medicines, Institute of Materia Medica, Chinese Academy of Medical Sciences/Peking Union Medical College, Beijing 100050, China

* Corresponding authors:

Dr Yan Wang, e-mail address: [wangyan@imm.ac.cn](mailto:wangyan@imm.ac.cn);

Dr Jian-dong Jiang, e-mail address: jiang.jdong@163.com.

**Supplementary Material**

**Investigation of composition of Total glucosides of white paeony capsules and Tripterygium wilfordii polyglycoside tablets.**

**Liquid Chromatography conditions for Total glucosides of white paeony capsules**

Analysis was performed using a liquid chromatography equipped with Photo-Diode Array detector (Shimadzu, Japan). Separation was performed using a C18 column (250 mm × 4.6 mm × 5 μm, Alltima, America). The flow rate was 1mL/min, and the column temperature was maintained at 30 °C. The mobile phases were phosphate: water (0.1:100, v/v) as mobile phase A and acetonitrile as mobile phase B. The binary gradient elution conditions are as follows (A:B): 0.01 min-20 min, 95:5→80:20; 20.01min-30min, 80:20→75:25; 30.01min-40min, 75:25→70:30; 40.01min-50min, 70:30→65:35; 50.01min-60min, 65:35; 60.01min-70min, 95:5. The detection wavelength is 230nm.

**Preparation of calibration standards and sample preparation of Total glucosides of white paeony capsules**

Stock solutions of paeoniflorin and albiflorin std were prepared by methanol individually in a final concentration of 3 mg/mL, while benzoylpaeoniflorin stock solution was prepared at 0.1 mg/mL. Stock solutions of paeoniflorin, albiflorin std and benzoylpaeoniflorin were combined and serially diluted with methanol to generate the calibration standards. The final concentrations of paeoniflorin and albiflorin std in the mixed standards were as follows: 1mg/ml, 0.5mg/ml, 0.25mg/ml, 0.125mg/ml, 0.0625mg/ml. The final concentrations of benzoylpaeoniflorin in the mixed standards were as follows: 0.1mg/ml, 0.05mg/ml, 0.025mg/ml, 0.005mg/ml, 0.0025mg/ml. For Total glucosides of white paeony capsules, the contents of the capsule were weighed and dissolved with methanol at 1mg/ml, then filtered by microporous membranes (0.22um). The filtrate were analyzed according to the above liquid chromatography conditions.

**Liquid Chromatography conditions for Tripterygium wilfordii polyglycoside tablets**

Analysis was performed using a liquid chromatography equipped with Photo-Diode Array detector (Shimadzu, Japan). Separation was performed using a C18 column (250 mm × 4.6 mm × 5 μm, Alltima, America). The flow rate was 0.75mL/min, and the column temperature was maintained at 30 °C. The mobile phases were phosphate: water (0.1:100, v/v) as mobile phase A and acetonitrile as mobile phase B. The binary gradient elution conditions are as follows (A:B): 0.01min-15min, 90:10→65:35; 15.01min-25min, 65:35→65:35; 25.01min-30min, 65:35→45:55; 30.01min-35min, 45:55→45:55; 35.01min-40min, 45:55→40:60; 40.01min-70min, 40:60→15:85; 70.01min-75min, 15:85→5:95; 75.01min-80min, 5:95→0:100, 80.01min-90min, 90:10→90:10. The detection wavelength for triptolide, celastrol, wilforine, wilforlide A and triptonide is 220nm, 425nm, 230nm 210nm and 220nm respectively.

**Preparation of calibration standards and sample preparation of Tripterygium wilfordii polyglycoside tablets.**

Stock solutions of triptolide, wilforine, wilforlide A and triptonide were prepared by methanol individually in a final concentration of 0.32 mg/mL, while celastrol stock solution was prepared at 0.01 mg/mL. Stock solutions of triptolide, celastrol, wilforine, wilforlide A and triptonide were combined and serially diluted with methanol to generate the calibration standards. The final concentrations of triptolide, wilforine, wilforlide A and triptonide in the mixed standards were as follows: 0.08mg/ml, 0.04mg/ml, 0.01mg/ml, 0.005mg/ml, 0.001mg/ml. The final concentrations of celastrol in the mixed standards were as follows: 0.01mg/ml, 0.005mg/ml, 0.001mg/ml, 0.0005mg/ml, 0.0001mg/ml. For Tripterygium wilfordii polyglycoside tablets, first crushed the tablets with a mortar, then weighed them, added methanol at 1mg/ml, dissolved them by ultrasound, then filtered by microporous membranes (0.22um). The filtrate were analyzed according to the above liquid chromatography conditions.

**Supplemental tables.**

Supplemental table 1 Concentration of each active ingredient in Total glucosides of white paeony capsules (10ug/ml, 100ug/ml)

| Total glucosides of white paeony capsules | 10ug/ml | 100ug/ml |
| --- | --- | --- |
| paeoniflorin | 6.03ug/ml | 60.3ug/ml |
| albiflorin std | 2.83ug/ml | 28.3ug/ml |
| benzoylpaeoniflorin | 0.083ug/ml | 0.83ug/ml |

Supplemental table 2 Concentration of each active ingredient in Tripterygium wilfordii polyglycoside tablets (10ug/ml, 100ug/ml)

| Tripterygium wilfordii polyglycoside tablets | 10ug/ml | 100ug/ml |
| --- | --- | --- |
| triptolide | 0.118ug/ml | 1.18ug/ml |
| celastrol | 0.0054ug/ml | 0.054ug/ml |
| wilforine | 0.52ug/ml | 5.2ug/ml |
| wilforlide A | 0.192ug/ml | 1.92ug/ml |
| triptonide | 0.088ug/ml | 0.88ug/ml |

Supplemental table 3. P values of differential metabolites compared with the control group after incubation with drugs or compounds

| Drug or compounds added | Metabolites | P values |
| --- | --- | --- |
| triptolide-L | Ipa | 0.003145 |
| triptolide-L | Phe | 0.0261663 |
| celastrol-L | Trp | 0.0074819 |
| celastrol-H | Iaa | 0.0002934 |
| celastrol-H | Pea | 0.0317665 |
| celastrol-H | Phe | 2.587E-05 |
| wilforine-L | Ipa | 0.0372448 |
| wilforine-L | Trp | 0.0264348 |
| wilforine-H | Ipa | 0.0348805 |
| wilforine-H | Pea | 0.0252672 |
| wilforine-H | Trp | 0.0088054 |
| wilforlide A-L | Pea | 0.024895 |
| wilforlide A-L | Phe | 0.0041069 |
| wilforlide A-H | Trp | 0.0004069 |
| wilforlide A-H | Phe | 0.006571 |
| triptonide-H | Phe | 0.004324 |
| tripterygium glycosides -L | Trp | 0.0130716 |
| tripterygium glycosides-H | Pea | 0.0385959 |
| paeoniflorin-L | Pea | 0.0340758 |
| paeoniflorin-L | Trp | 0.0332624 |
| paeoniflorin-L | Phe | 0.0241918 |
| paeoniflorin-H | Trp | 0.0023028 |
| paeoniflorin-H | Phe | 0.0139379 |
| albiflorin std-L | Trp | 0.0013393 |
| albiflorin std-H | Pea | 0.0274233 |
| albiflorin std-H | Trp | 0.0014718 |
| benzoylpaeoniflorin-L | Pea | 0.0482403 |
| benzoylpaeoniflorin-L | Phe | 0.0007892 |
| benzoylpaeoniflorin-H | Trp | 0.0240685 |
| total glucosides of paeony-L | Phe | 0.0002536 |
| total glucosides of paeony-H | Trp | 0.0021219 |
| total glucosides of paeony-H | Phe | 0.0124558 |

**Supplemental figures.**

**Figure S1**

**
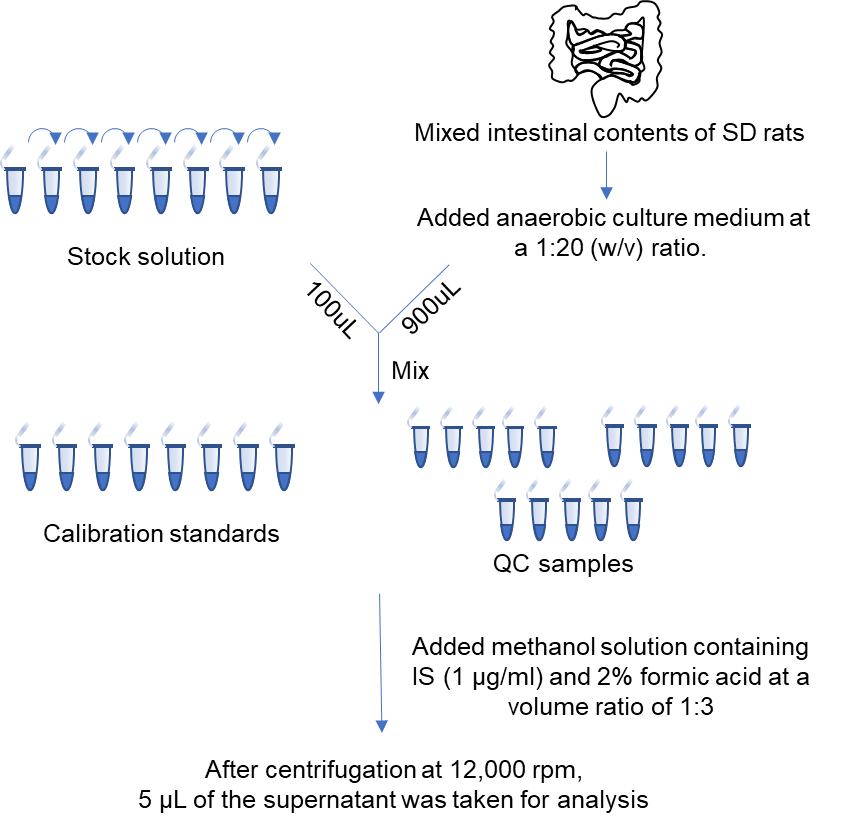
**

**Figure S2**

**
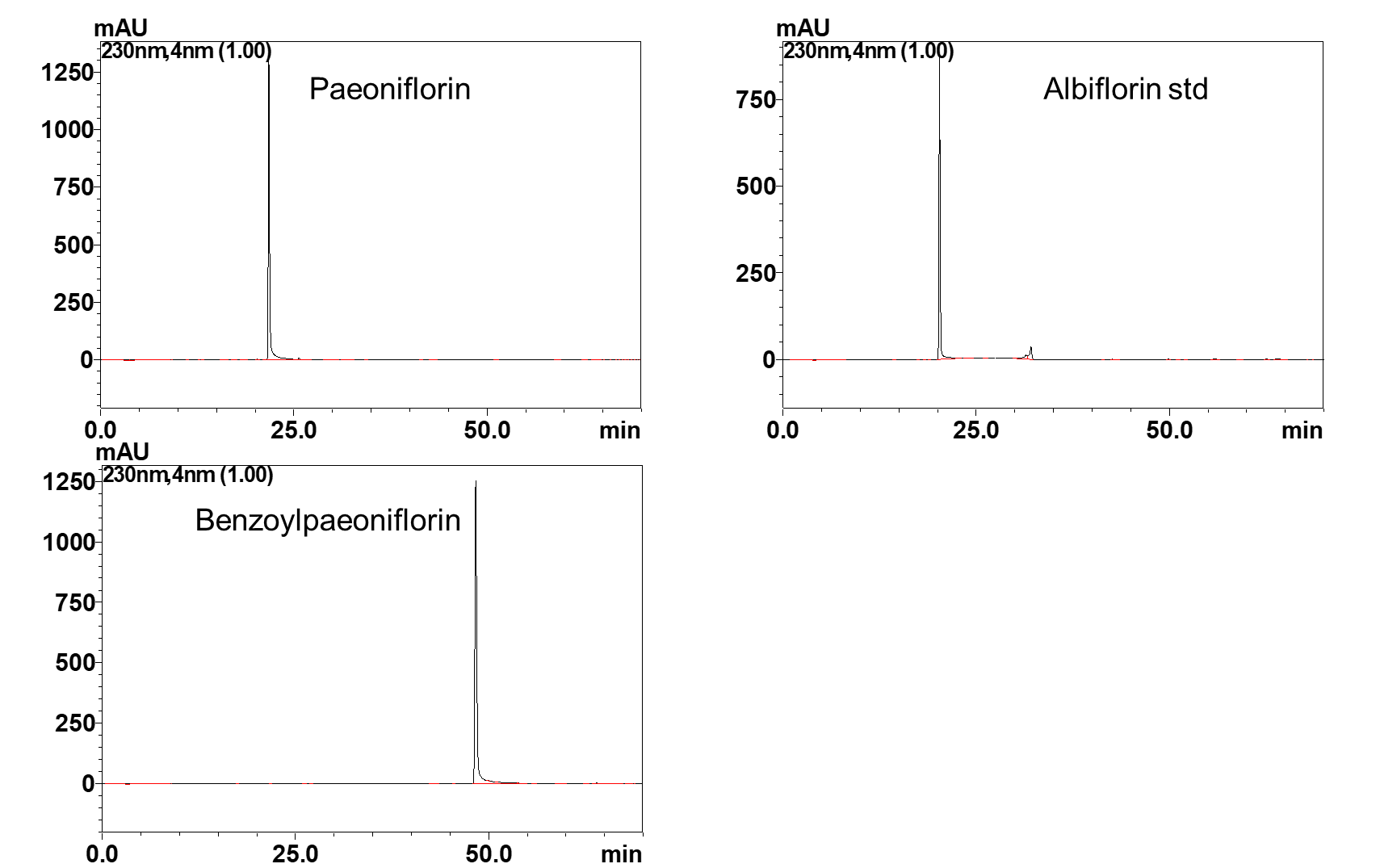
**

**Figure S3**

**
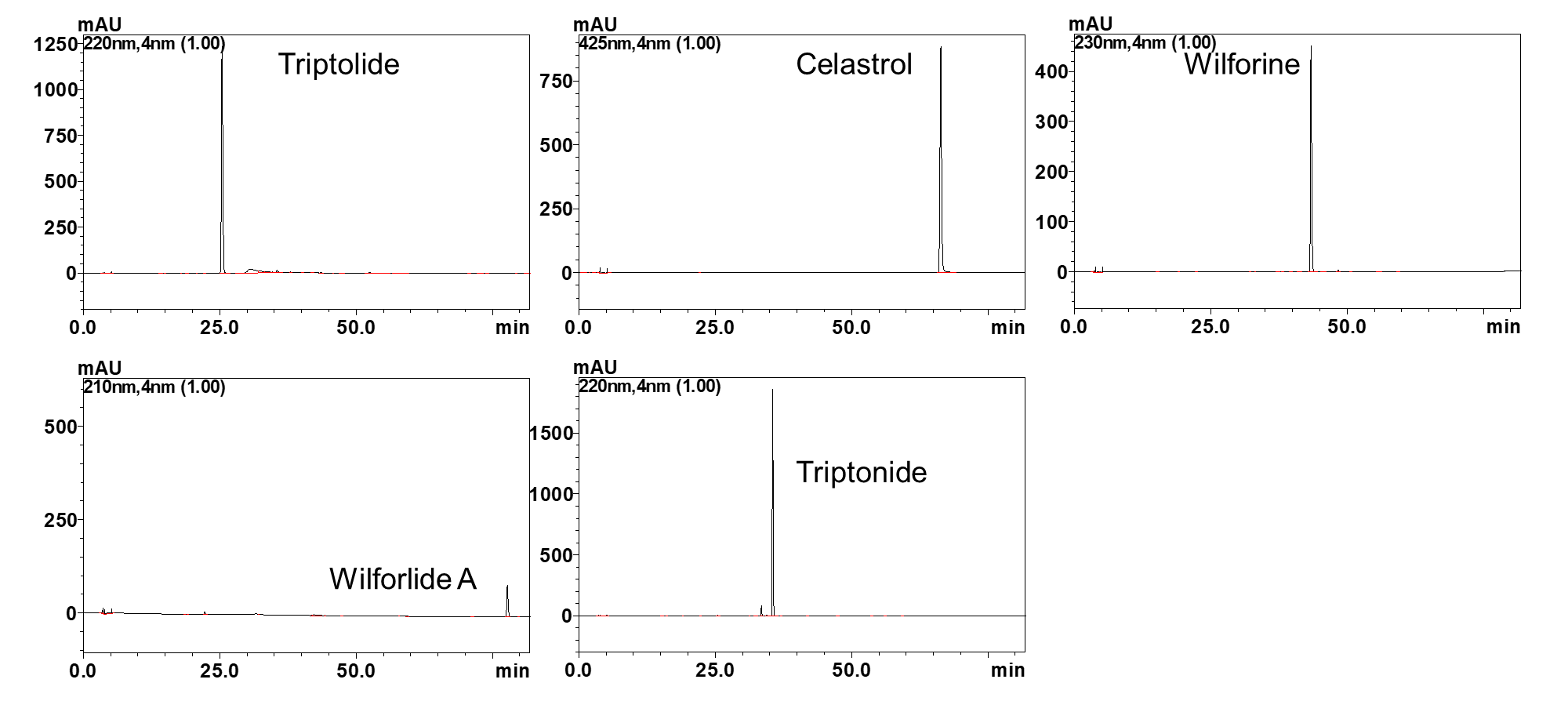
**

**Figure legends**

Figure S1: The process of preparation of calibration standards and QC samples.

Figure S2: The liquid chromatography spectrum of paeoniflorin, albiflorin std and benzoylpaeoniflorin.

Figure S3: The liquid chromatography spectrum of triptolide, celastrol, wilforine, wilforlide A and triptonide.
